# Closing the Gap on COVID-19 Vaccinations in First Responders and Beyond: Increasing Trust

**DOI:** 10.3390/ijerph19020644

**Published:** 2022-01-06

**Authors:** Megan E. Gregory, Sarah R. MacEwan, Alice A. Gaughan, Laura J. Rush, Jonathan R. Powell, Jordan D. Kurth, Eben Kenah, Ashish R. Panchal, Ann Scheck McAlearney

**Affiliations:** 1 Department of Biomedical Informatics, College of Medicine, The Ohio State University, Columbus, OH 43210, USA; ann.mcalearney@osumc.edu; 2 The Center for the Advancement of Team Science, Analytics, and Systems Thinking in Health Services and Implementation Science Research (CATALYST), College of Medicine, The Ohio State University, Columbus, OH 43210, USA; sarah.macewan@osumc.edu (S.R.M.); alice.gaughan@osumc.edu (A.A.G.); laura.rush@osumc.edu (L.J.R.); 3 National Registry of Emergency Medical Technicians, Columbus, OH 43210, USA; jpowell@nremt.org (J.R.P.); jkurth@nremt.org (J.D.K.); Ashish.Panchal@osumc.edu (A.R.P.); 4 Division of Epidemiology, The Ohio State University College of Public Health, Columbus, OH 43210, USA; 5 Division of Biostatistics, The Ohio State University College of Public Health, Columbus, OH 43210, USA; kenah.1@osu.edu; 6 Department of Emergency Medicine, The Ohio State University Wexner Medical Center, Columbus, OH 43210, USA; 7 Department of Family and Community Medicine, College of Medicine, The Ohio State University, Columbus, OH 43210, USA

**Keywords:** COVID-19, vaccine hesitancy, frontline healthcare workers, emergency medical services, medical mistrust

## Abstract

Although COVID-19 vaccines are widely available in the U.S. and much of the world, many have chosen to forgo this vaccination. Emergency medical services (EMS) professionals, despite their role on the frontlines and interactions with COVID-positive patients, are not immune to vaccine hesitancy. Via a survey conducted in April 2021, we investigated the extent to which first responders in the U.S. trusted various information sources to provide reliable information about COVID-19 vaccines. Those vaccinated generally trusted healthcare providers as a source of information, but unvaccinated first responders had fairly low trust in this information source—a group to which they, themselves, belong. Additionally, regardless of vaccination status, trust in all levels of government, employers, and their community as sources of information was low. Free-response explanations provided some context to these findings, such as preference for other COVID-19 management options, including drugs proven ineffective. A trusted source of COVID-19 vaccination information is not readily apparent. Individuals expressed a strong desire for the autonomy to make vaccination decisions for themselves, as opposed to mandates. Potential reasons for low trust, possible solutions to address them, generalizability to the broader public, and implications of low trust in official institutions are discussed.

## 1. Introduction

### 1.1. COVID-19 Vaccinations in the U.S.

Leading public health organizations agree that vaccination is a critical strategy to combat the COVID-19 pandemic [1,2]. However, in spite of a massive vaccine campaign, as of early October 2021, only 65.1% of those eligible in the U.S. have been fully vaccinated [3]. That proportion is less for individuals aged 18–24 (52.1%) and 25–39 (56.2%) [4]. The emergence of the COVID-19 variants have added greater urgency for vaccine protection. The Delta variant is more contagious than earlier strains of COVID-19 [5] and at the time of this writing, accounted for most new COVID-19 cases in the U.S. [6]. Importantly, the currently available vaccines provide significant protection against serious consequences of COVID-19 infection [7].

There are many reasons why people might choose to forgo COVID-19 vaccination. One of these factors involves trust. To understand the impact of trust, a greater understanding of the interaction between trust and vaccine hesitancy is necessary. A specific population where this interaction is critical to public health is first responders. These individuals are uniquely situated to be at increased risk for exposure, and their decisions to receive a vaccination impact not only their health and safety but also the health and safety of their patients. In this article, we examine the relationship between COVID-19 vaccine hesitancy and trust within an important group of first responders—emergency medical service (EMS) professionals—and discuss approaches to building trust that may help strengthen our response to the COVID-19 pandemic as well as healthcare infrastructure.

### 1.2. Vaccine Hesitancy

Vaccine hesitancy is the delay or refusal to get a vaccine when it is available [8]. The first vaccines against SARS CoV-2 (COVID-19) became available in December 2020 through Emergency Use Authorizations (EUAs) by the U.S. Food and Drug Administration (FDA). Currently, there are three vaccines available in the U.S., two for adults 18 and older and one for children ages 5 and older [9]. Mass vaccination sites and pop-up clinics to administer the vaccine free of charge have been rapidly established in traditional healthcare facilities, community centers, sports arenas, shopping mall parking lots, and many other locations in an attempt to provide easy access while maintaining social distancing. Some states even implemented door-to-door visits by public health workers to offer vaccines to people in their homes [10]. Yet, even with these options, many individuals who could receive a vaccine remain unvaccinated.

The underlying reasons for vaccine hesitancy are multifactorial and complex. The World Health Organization (WHO) EURO Vaccine Communications Working Group proposed that trust in the safety and efficacy of a vaccine, the health system providing it, and policymakers advocating for it are important determinants of vaccine hesitancy [11]. However, survey data show a decades-long decline in trust of medical leaders in the U.S. [12], which could contribute to COVID-19 vaccine hesitancy. In fact, the erosion of public trust in science has become so profound that in 2017, the National Academy of Sciences organized a workshop to understand the sources of mistrust and explore opportunities for government, university, and industry partner collaborations to reverse this trend [13].

### 1.3. Assessing Vaccine Hesitancy among Healthcare Workers and First Responders

COVID-19 vaccine hesitancy among healthcare workers has not been extensively studied. A survey by the Kaiser Family Foundation in December 2020 indicated that close to one-third of healthcare workers in the U.S. were unlikely to get the COVID-19 vaccination [14]. Berry et al. reported that misinformation, especially from social media, and concern about side effects played an important role in vaccine hesitancy in workers in skilled nursing facilities [15]. One category of healthcare workers about which we currently lack insight is first responders. Notably, first responders are at personal risk for COVID-19 infection through workplace exposure as they interact with a cross-section of the public, frequently under emergent, uncontrolled, and chaotic conditions. Additionally, as first responders are at high risk for exposure, they may also subsequently transmit the virus to patients, co-workers, household contacts, and emergency department staff during patient transfer activities.

In spite of their importance as first responders, the extent of vaccine hesitancy among the more than 1 million EMS professionals in the U.S. [16] is largely unknown. Two studies conducted prior to the availability of COVID-19 vaccines indicated that uptake of the vaccine after approval might be less than ideal in this group. A German study found that just over half (57%) of EMS professionals surveyed responded in favor of receiving the vaccine [17], and in the U.S., fewer than half (48.2%) of first responders indicated a strong preference for the vaccine [18]. With the release of COVID-19 vaccines in the U.S., it is still unknown whether EMS professionals are likely to be vaccinated even though they are at high risk for infection. Understanding vaccine hesitancy among EMS professionals and how to help these first responders overcome hesitancy is important in providing a work environment that is safe for the EMS workforce as well as for patients and others with whom they may come in contact.

### 1.4. Assessing EMS Vaccine Hesitancy

To fill the gap, we sought to gain insight about the factors that influence a person’s decision whether or not to receive a COVID-19 vaccine using a representative sample of EMS professionals. We developed and distributed a survey to address these questions, which sought to assess COVID-19 vaccination perceptions among EMS professionals across the U.S. As a part of this survey, we asked questions about whom respondents trusted regarding COVID-19 information. We recognize that trust is complicated, multifactorial, and may vary both within and between persons. As such, we examined trust in information from numerous sources within the multiple domains of healthcare, government, media, and one’s community. From here, we identified themes.

## 2. Materials and Methods

### 2.1. Participants and Procedure

We distributed a survey to a simple random sample of civilian EMTs and paramedics aged 18–85 years old from a database of the National Registry of Emergency Medical Technicians (NREMT), the national certification body for EMS in the U.S. Survey distribution began on 20 April 2021, which was after vaccinations had been released to all Centers for Disease Control and Prevention (CDC) phase 1a and 1b groups (including EMS professionals) [19,20,21] and after the Department of Health and Human Services had released a directive on 17 March 2021 to vaccinate all adults due to sufficient vaccine supply [22]. Thus, all EMS professionals had had an opportunity to be vaccinated prior to study participation.

### 2.2. Measures

#### 2.2.1. COVID-19 Vaccination Status

We asked participants whether they had been vaccinated against COVID-19, with dichotomous response options (yes/no).

#### 2.2.2. Trust in Information Sources

We provided a list of information sources (e.g., federal government, state government, local government, doctors and other healthcare professionals, family and friends, a religious leader or organization, one’s employer, TV/radio/newspaper, medical websites, search engines, social media) and asked participants to check-all-that-apply to indicate which of these sources they trusted to provide reliable information about COVID-19 vaccines. Responses were scored dichotomously for each source, and a sum of the number of sources trusted was computed.

#### 2.2.3. Open-Ended Comments

Five open-ended questions were asked in the survey. For those who were vaccinated against COVID-19, we allowed them to indicate why they chose to be vaccinated; they could select all that apply amongst a list of options and/or select “I got it for another reason” and provide an open-ended response with more explanation. We then asked these participants if they got the vaccine as soon as they were eligible or if they waited. If they waited, they were asked to indicate why by selecting all that apply from a list of options and/or by selecting “I waited for another reason” and providing an open-ended response with more explanation. Similarly, for those unvaccinated, we allowed them to indicate why they chose not to be vaccinated by selecting all answers that apply from a list of options and/or by selecting “I do not want to receive a vaccine for another reason” and providing an open-ended response with more explanation. We then asked these participants whether or not they planned to get the vaccine in the future. If they said they were planning to get it in the future, they were asked to indicate why they had not received it yet by selecting all answers that apply from a list of options and/or by selecting “I am waiting for another reason” and providing an open-ended response with more explanation. Finally, we asked all participants the following question: “What else would you like to share regarding your thoughts about or experiences with COVID-19 vaccines?”

### 2.3. Analysis

Results were analyzed in two ways: we analyzed quantitative data using IBM SPSS Statistics and we coded and analyzed open-ended comments using ATLAS.ti. For quantitative data, missing data were dropped listwise. Descriptive statistics were computed to determine the percentage of respondents who trusted each information source. We stratified this by the individual’s self-reported COVID-19 vaccination status and computed Chi-square tests to compare the proportions of those who did and did not trust each information source by vaccination status. This test was chosen as it is the appropriate statistical test for testing two categorical variables. Then, we reviewed and coded the respondents’ open-ended comments. Comments specifically related to the domains included in our trust measure were of particular interest.

## 3. Results

Overall, 2257 survey participants reported their COVID-19 vaccination status and responded to the items on sources of trust. Table 1 provides the demographics of the participants.

Below, we discuss findings from the measures of trust and present the major themes we characterized from the open-ended comments. Representative comments are presented both in text as well as in figures.

### 3.1. Theme 1: Low Trust in Government

**Figure 1:**
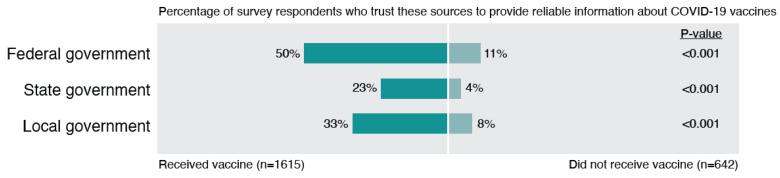
Differences in trust in government between vaccinated and unvaccinated EMS professionals.

**Figure 2:**
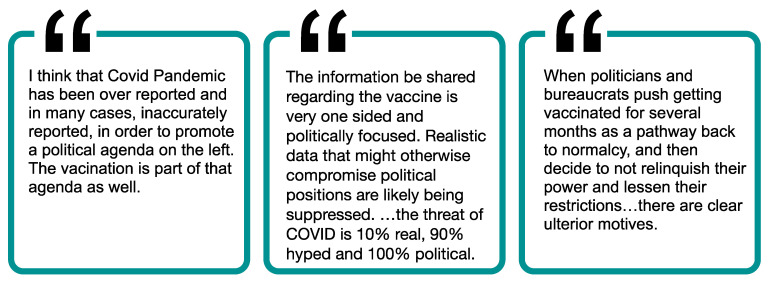
Participant perspectives regarding mistrust in government.

Trust in all levels of government (federal, state, local) was low (Figure 1), and mistrust in the government was a very common theme among the open-ended responses (Figure 2). Although vaccinated individuals had significantly higher rates of trusting the government, it is interesting that trust was fairly low even among those who were vaccinated. As shown in the open-ended responses, some individuals had more moderate concerns about how the government handled COVID-19 vaccines, while others expressed more strongly worded opinions, such as that they felt they had been lied to, that the government was withholding effective treatments, or that the COVID-19 situation was a political weapon. For example, one respondent noted, “I genuinely feel that it is… a political weapon. You didn’t see this sh*t go down with SARS, the common cold, the flu, etc.” Similarly, another respondent shared, *“Politics played a MAJOR role in this pandemic. I don’t trust our government and it’s* [sic] *involvement in this vaccine development. Although ‘experts’ claim the speed of development has nothing to do with efficacy or safety, it still does not negate the fact that no one knows what the long-term effects of the vaccine will be.”.*

Other sources of mistrust of the government included beliefs that the government has blown the pandemic out of proportion, that the government was lying about the origin of COVID-19, the number of cases, or the severity of disease, and that political pressure rushed the development of the vaccine. Furthermore, individuals expressed frustration with government guidelines and restrictions that frequently changed throughout the pandemic and were related to the vaccine (e.g., whether or not masks were still mandatory after vaccination). Individuals were also wary about how hard the government was promoting the vaccine. Ultimately, the politicization of the pandemic and the COVID-19 vaccine made it difficult for some individuals to know what to believe, as described by one respondent: *“COVID information and the vaccines were such a political issue that I never really knew what or who to believe. And to be honest, with all the misinformation/disinformation, I lost trust in our institutions.”*

### 3.2. Theme 2: Mistrust in Healthcare and Medical Sources

**Figure 3:**
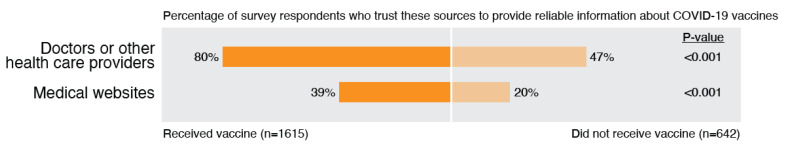
Differences in trust in healthcare and medical sources between vaccinated and unvaccinated EMS professionals.

**Figure 4:**
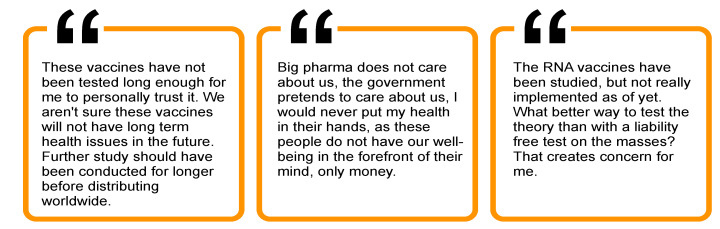
Participant perspectives regarding mistrust in medical community.

A significant amount of mistrust in healthcare and medical sources was found in the survey data (Figure 3), and another common theme was observed across the open-ended responses (Figure 4), especially among those who were unvaccinated. It is troubling that half of the unvaccinated individuals do not trust doctors, and 80% do not trust legitimate medical websites. It is also notable that 20% of individuals who received a vaccine reportedly do not trust doctors and 60% of them do not trust medical websites, although they trusted these sources at higher rates than unvaccinated individuals.

In our survey, we did not explicitly ask about trust in pharmaceutical companies or scientists/researchers, but we did find that trust in healthcare and in medical sources of information were common topics discussed in open-ended responses. Many participants commented that the development of the vaccine by pharmaceutical companies was rushed and that more research needed to be done, stating concerns about unknown short- and long-term side effects of the vaccine. For example, one respondent noted, “*When people say ‘I’ve done my research’ they are misinformed. The vaccine recipients ARE infact* [sic] *the research. I am awaiting those results. There will be long-term effects. There will be cancer. There will be sterilization of once fertile men and women. There WILL be a remarkable spike in birth defects.”* Others similarly shared, *“Not enough research has been conducted due to not enough time having passed to assess risk of long-term side effects.”*

Some participants highlighted mistrust in the new mRNA vaccine technology, specifically. One participant shared, “*It is unfortunate that we have so little information about the mRNA technology they are implementing. The smartest physicians I know only can explain that it teaches the body to produce a spikey protein… we know the science is far more complicated than that and I’m sure it is highly secretive. We don’t really have a clue what we are injecting ourselves with. Just have faith. I have zero faith in government and big pharma.*” Concerns seemed to be heightened by the fact that the vaccines were not FDA approved (at the time of the survey) and that pharmaceutical companies would not be held accountable for the consequences of the vaccine when used under emergency authorization. Some participants pointed out their mistrust in pharmaceutical companies, noting that these companies were motivated only by money. Others were reportedly frustrated that there was not more information about the efficacy of the vaccine, how long immunity would last, and how vaccination affects the transmission of the disease. Furthermore, a perception that the data and science surrounding the vaccines kept changing also contributed to a lack of trust in the vaccine research.

### 3.3. Theme 3: Low Trust in Media

**Figure 5:**
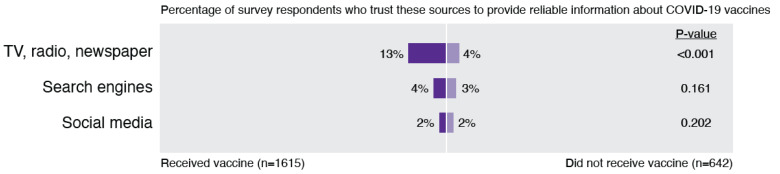
Differences in trust in media between vaccinated and unvaccinated EMS professionals.

Trust in media sources was notably low for both vaccinated and unvaccinated individuals, although those vaccinated respondents had significantly higher rates of trust in TV, radio, and newspaper sources (Figure 5). In addition, consistent with prior work that suggested social media as a major source of COVID-19 vaccine misinformation [15], only 2% of unvaccinated and 1.5% of vaccinated individuals endorsed this as a source they trust.

**Figure 6:**
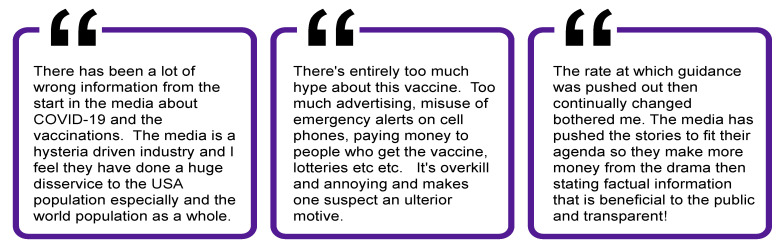
Participant perspectives regarding mistrust in media.

In open-ended responses, participants described the impact of media on their perspectives about the pandemic in general and the vaccine specifically (Figure 6). Some participants noted that they felt the media blew the pandemic out of proportion and were responsible for using scare tactics and spreading fear. With regard to the vaccine, some participants were reportedly not satisfied with the information shared in the media, with one participant offering, *“I’m pro-vaccinations, with my most recent one being an annual flu shot last fall. But the public-communication effort for COVID-19 has been a disaster. As there are for all such things, there are serious questions about safety and efficacy that deserve to be addressed and that have nothing to do with any anti-vaxxer sentiment. The media effort to address those questions has been undermined by the failure to acknowledge many of them and by the transparent and admitted dishonesty of Dr. Fauci and others whom the media consistently cite for the information they provide. An apparent lack of concern for credibility is the biggest failure of the COVID-19 vaccination education campaign.”*

Some participants also felt suspicious of the hype and pressure for vaccination pushed by the media. Several pointed out their frustration with the role of social media in spreading misinformation and conspiracy theories. Participants also expressed frustration that media sources shared conflicting information and guidance, which led to confusion; as one respondent simply noted, *“Media has made it impossible to tell what to believe and not believe.”* Mixed and changing media messaging around COVID-19 has appeared to lead to significant confusion and mistrust.

### 3.4. Theme 4: Low Trust in Employer and Community

**Figure 7:**
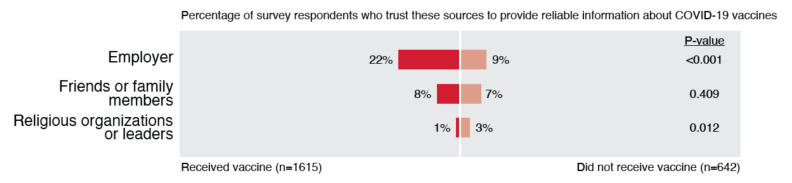
Differences in trust in employer and community between vaccinated and unvaccinated EMS professionals.

Individuals’ trust in their employers and communities—including their friends, families, and religious groups—was also low (Figure 7). It is curious that so few indicated they trusted their employer, as the sample was composed entirely of EMS professionals. It is also interesting that so few indicated they trusted friends or family, as prominent social psychology theories posit that individuals have high trust in these types of ingroups [23]. While very few respondents reported trusting religious organizations or leaders, this trust was significantly higher in the unvaccinated group compared to the vaccinated group.

**Figure 8:**
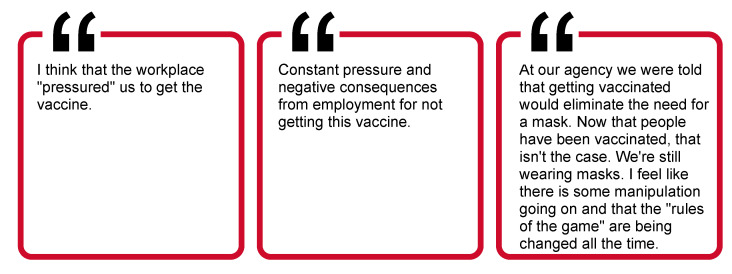
Participant perspectives regarding mistrust in employer and community.

In the open-ended comments, some participants did provide examples of how their community influenced their perspectives about the vaccine (Figure 8). Many participants described how they were influenced by the experiences of family and friends, including experiences with severe illness from COVID-19, experiences with bad side effects from the vaccine, or experiences getting COVID-19 after being vaccinated. For example, one participant explained, *“I had the Pfizer vaccine and had no side effects. However, I had co-workers who were debilitated for 24–47 hours post the 2nd dose. While I don’t believe there is some grand conspiracy there is just enough truth in both pro and con views that neither can be fully trusted.”* Participants also mentioned the role of their employer and were concerned about their employer pressuring them to get vaccinated and implementing negative consequences for those who were not vaccinated. Changing guidelines from employers related to the vaccine (e.g., the decision to require masks regardless of vaccination status) were another reported source of frustration for participants, echoing the frustration noted with the changing information shared by the government, medical community, and media. Although no respondents mentioned religious organizations or leaders in their comments, some did reflect a trust in God to protect them from COVID-19. For example, one participant shared, *“It is not against my religion however I believe that God will protect me. I also believe that if I do acquire the disease that it is meant to be.”* Finally, many participants commented that it was an individual’s personal choice to receive the vaccine, which may help explain low trust, even in their communities. As one respondent shared, *“It should be a personal choice to whether or not you get the vaccine. Just like the flu vaccine. … It’s your choice, and yours alone. Friends, family, coworkers, employers, and government should have no say in your immunizations.”*

### 3.5. Theme 5: Many Trust No One

**Figure 9:**
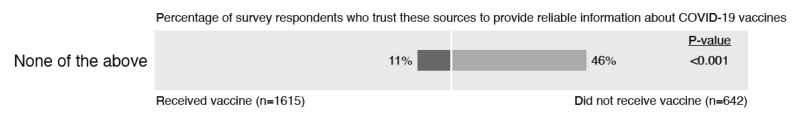
Differences in trust in no sources between vaccinated and unvaccinated EMS professionals.

Almost half of the respondents who were not vaccinated did not endorse any of the listed sources as ones they trusted to provide reliable information about COVID-19 vaccines (Figure 9). It is unclear who, if anyone, these individuals trust or where they obtain their information. Further, the average number of sources that both vaccinated and unvaccinated individuals reported trusting was low: only 1.2 of 11 for those who were unvaccinated (SD = 1.66, Mdn = 1.00), and 2.7 of 11 for those who were vaccinated (SD = 1.95, Mdn = 2.00), with those who were vaccinated trusting a greater number of information sources (*p* < 0.001). Of note, while there were significantly fewer people in the vaccinated group who trust no one and a higher number of sources trusted, trusting only 1–2 sources is troublesome. Respondents’ open-ended comments shed additional light on this lack of trust across all sources. One participant shared, *“Extreme amount of conflicting behavior* [sic]*/ information from all sources. Led to issues of trusting any source.”* Another respondent similarly noted, *“Everyone is guessing about all of it. There are way too many unknowns over the last year to trust any source of information completely. At the rate that information changes, it’s easy to see why trust is low.”*

### 3.6. Additional Noteworthy Themes

#### 3.6.1. Some Individuals Trust in Other Options to Manage COVID-19 Rather Than the Vaccine

**Figure 10:**
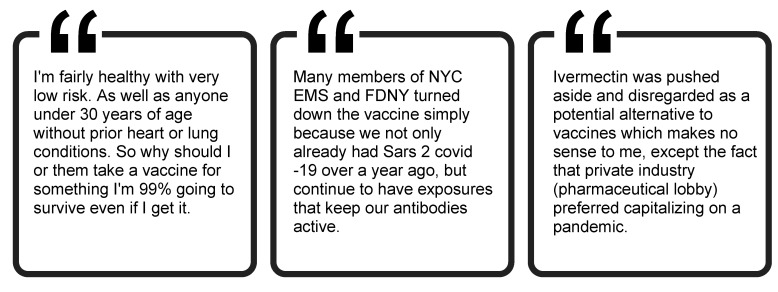
Participant perspectives regarding trust in other options to manage COVID-19.

While not related to trust in information sources—and generally not indicating where their information came from—many participants indicated they trusted things other than the vaccine to get them through COVID-19 (Figure 10). Such sources included God, one’s own immune system or current health status, the high survival rate among those who contracted COVID-19, antibodies from prior infection, and medication—both medications shown to be effective for the treatment of COVID-19 (e.g., Remedesivir) [24] and those that are not (e.g., hydroxychloroquine). Other comments suggested skepticism related to having had multiple COVID-19 exposures over the course of the pandemic yet not acquiring the disease themselves.

#### 3.6.2. Many Argue for the Autonomy to Decide for Themselves

**Figure 11:**
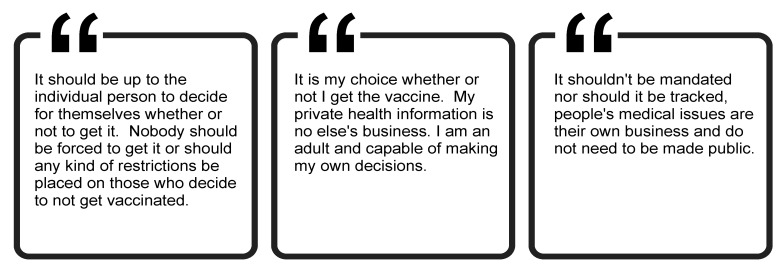
Participant perspectives regarding autonomy to decide about vaccination.

Others indicated the desire to have the autonomy to decide for themselves whether to get a COVID-19 vaccine and were strongly against mandates (Figure 11). Respondents stressed that the choice to be vaccinated was a personal one and that their decision to vaccinate or not should be private information. These quotations generally did not allude to trust or mistrust, reinforcing the suggestion that there are multiple layers to vaccine hesitancy, and increasing trust will not increase vaccination rates on its own.

#### 3.6.3. Negative Experiences with the Vaccine May Reduce Willingness for Booster Shots

**Figure 12:**
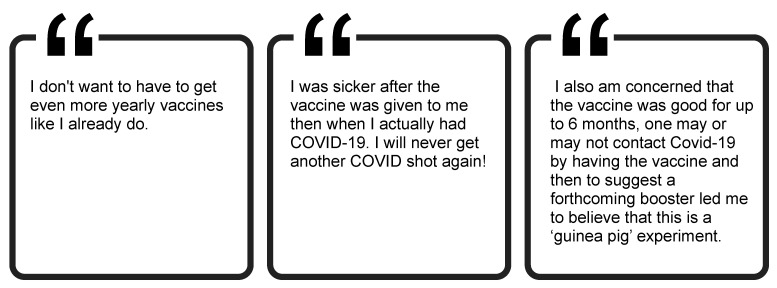
Participant perspectives regarding booster shots.

Lastly, while we did not explicitly ask about booster shots, some comments discussed this topic (Figure 12). Among those who were vaccinated, some respondents said the side effects that they experienced after getting the vaccine convinced them not to get the second dose of their vaccine or to decide not to get another COVID-19 shot in the future. Another respondent expressed post-vaccine regret, citing persistent concerns about vaccine safety despite their decision to be vaccinated. Others were more explicit about their concerns around booster shots. Some simply did not want to have to get the shot on a yearly (or more frequent) basis. Finally, others stated that the conversation around the necessity for boosters made them more hesitant about the vaccine. It is likely that all of these sentiments may cause individuals to be more resistant to receiving booster shots, as recommendations for these boosters emerge.

## 4. Discussion

Overall, vaccinated individuals had significantly higher rates of trust in almost every source of information. These findings are in line with prior conceptual work that has posited a relationship between factors such as trust in healthcare providers, trust in the pharmaceutical industry, the media environment, and vaccine hesitancy [11]. Regardless, trust in information sources was generally fairly low for both the unvaccinated and vaccinated groups in our study. The findings of this study beg two questions: why do individuals not trust information sources such as healthcare providers and government, and whom *do* they trust on the issue of COVID-19 vaccines?

### 4.1. Reasons for Low Trust

Toward the first question, we look to the quotations around low trust across sources (i.e., trusting no one). A major theme is mistrust due to COVID-19 information that has changed over time. For example, early in the pandemic, in an effort to manage the shortage of personal protective equipment (PPE), the federal government advised that face masks were unnecessary and ineffective for protecting against COVID-19 [25]. Weeks later, the Centers for Disease Control reversed course and advised that all Americans wear masks in public [26]. Over the course of the pandemic, the number of face masks that the U.S. government recommended the public to wear in public ranged from zero [25] to two [27], with officials often flip-flopping on their own advice [28,29]. In addition, the unprecedented fast pace of research on COVID-19 led to beneficial breakthroughs but also led to non-peer-reviewed pre-print articles making rounds in the media, which often later proved to contain errors and unfounded conclusions [30]. This particular issue, along with in vitro studies showing potential effectiveness [31], contributed to individuals trusting in hydroxychloroquine as a potential treatment; yet, as more data came out indicating this drug was ineffective [32], many members of the public were resistant to this changing information, contributing to mistrust. Instead of being open to changing data in light of the scientific process, confirmation bias may have led those who were predisposed to have doubt in pharmaceutical, medical, or government institutions to further their mistrust due to these changes.

These types of changing messages as the pandemic evolved appeared to lead to an erosion of trust in institutions for many respondents, indicating that leadership in a public health crisis needs to send messages that are internally consistent and that accurately communicate any potential uncertainties. Messaging should acknowledge that the scientific process is fluid and that information is subject to change in light of more data. While this may seem obvious to scientists and researchers, those outside of these professions are typically not as informed on the scientific process and historically receive information after studies have been complete and research questions have more definitive answers, rather than watching the science play out in real-time. Thus, the general public does not typically have a nuanced view of the fluid scientific process to which those in the field are accustomed, potentially sowing mistrust in the current fast-paced scientific climate of COVID-19. Furthermore, in addition to messaging about the realities of the scientific discovery process, more science education should be given. Considering our findings are from EMS professionals, whose profession requires them to obtain greater scientific and healthcare-related knowledge than the lay public, it is likely that a more scientifically naïve public would benefit even further. In the context of the current study, the EMS curriculum could be expanded to cover topics such as virology and immunology to increase understanding about the importance of vaccination and risks in this population, especially given the relevance of these topics to EMS professionals’ jobs.

### 4.2. Whom Do Individuals Trust?

Regarding whom individuals *do* trust, that question remains unanswered. Participants generally did not mention which alternative sources of information they trust and use. Yet they often mentioned information they believed to be true, such as drugs they believed were effective against COVID-19 and the idea that they did not need the vaccination due to prior infection or high survival rates. It is unclear where the participants received this information. In future work, we plan to examine this question in more depth.

Regardless, when considering the relatively low levels of trust among the vaccinated EMS professionals we surveyed, it is clear that trust alone is not the definitive driving factor in the decision to receive a vaccination. Other factors must have overcome these individuals’ lack of trust and made them decide to receive the vaccine. Our prior work with this population indicates that the most common reasoning given for getting the COVID-19 vaccine was to protect oneself and to protect others [33]. Only a small percentage indicated they were mandated to receive the vaccine by a job, family member, or for other activities [33]. Further, our prior work found that the receipt of the vaccine was highly correlated with the perceived risks of COVID-19 [33]. Thus, it is possible that messaging may have been enough to convey that COVID-19 was a threat and thus to convince these individuals to receive a vaccination. Reconciling this with the findings of low trust, even among the vaccinated, remains an issue to explore further in future research.

### 4.3. Major Concerns of the Vaccine-Hesitant

One noteworthy theme regarding vaccine hesitancy that came out in many of the respondents’ comments was concerns about unknown long-term side effects. While we highlighted only a few responses on this topic in this paper, the concern was widely noted across our dataset. Furthermore, despite the prevalence of this concern about long-term side-effects, this issue has not been commonly addressed in media or government information about the vaccine, which has tended to focus on myth-busting around topics such as microchips, fertility, and the impact of mRNA vaccines on DNA [34], as well as emphasizing general safety and short-term side effects [35]. Messaging should be more clear about potential long-term side effects, highlighting that these are very unlikely for known reasons such as that vaccines are eliminated from the body quickly, side effects tend to show up within weeks, and there have been no major long-term side effects noted beyond one month in those who have been vaccinated [36]. In addition, messaging regarding long-term issues should also clarify that COVID-19 infection itself is associated with long-term side effects for potentially 10–20% of those infected, with these long-term effects impacting quality of life and often including impairment in multiple organs [37,38,39]. Along these lines, messaging should be careful to establish that any risks of the COVID-19 vaccine must be considered in the context of known risks from COVID-19 infection. Specifically, recent work has demonstrated that compared to risks from COVID-19 vaccines, infection with the SARS-CoV-2 virus carries a higher risk for almost all severe outcomes studied, including myocardial infarction and deep vein thrombosis [40]. This is especially important given the recent consensus that the virus is likely to become endemic, making COVID-19 infection a likely alternative to vaccination [41]. Those individuals who are vaccine-hesitant should be informed about and encouraged to take these risks into account when making a decision about getting the vaccine.

In addition, many comments indicated that individuals wanted autonomy to decide for themselves about getting the vaccine rather than being mandated to do so. Given this sentiment, employer mandates for COVID-19 vaccination, which are increasingly common now that FDA approval of the Pfizer-BioNTech vaccine has been received [42], may be challenged by some first responders, as was the case for mandatory vaccinations against influenza that were not supported by EMS professionals [43]. Thus, while vaccination mandates align with healthcare workers’ ethical responsibilities to do no harm, they also may further weaken EMS professionals’ trust in healthcare systems [44]. Some states have alternatively tried incentivizing vaccines (e.g., Ohio’s vaccination lottery [45]; USD 100 payments in some Texas counties [46]), with mixed evidence about success [47,48]. While this approach may motivate some people and align with preferences for individual choice, those who remain hesitant due to safety concerns may not be swayed by these incentives, especially when those incentives come from sources that they do not trust.

### 4.4. Messaging to Improve Trust

**Figure 13:**
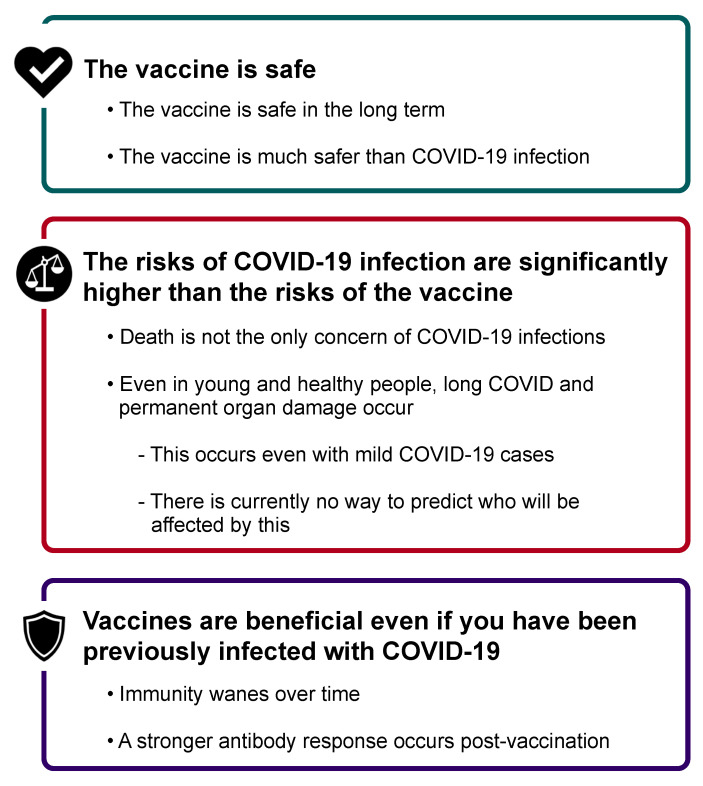
Recommended messaging around COVID-19 vaccination.

Based on our data, we recommend that future messaging around COVID-19 vaccinations focus on the three key areas identified in Figure 13. In addition, there would likely be a benefit in acknowledging previous mixed messaging by official sources, with rationale provided for these changing messages (e.g., better data as the scientific process evolves). Further, while we did not explicitly investigate political affiliations, it is likely that more bipartisan messaging could help, as many comments mentioned politicians they favored or disliked when commenting about the COVID-19 vaccines and the pandemic. However, as trust in government was low overall, non-political messaging would be helpful as well. Dr. Fauci, and other government officials who are known names in the COVID-19 arena, appeared multiple times in respondents’ comments and were often disparagingly referenced. Given low levels of trust in information coming from the government, media, community, or healthcare officials, it unfortunately remains unclear who and what might be the best sources of messages about COVID-19 and the importance of vaccinations.

### 4.5. Limitations

Despite these interesting findings, there are some limitations to our study. First, the study was focused on first responders (i.e., EMS professionals) and not the general public; although many of the results are consistent with discourse noted about the lay public, some caution should be warranted when attempting to generalize these findings. The second is related to our sample, recognizing that these perceptions are from individuals who work at the front lines and can interact with COVID-positive patients. Individuals who do not frequently see the realities of this disease up close may be even more wary and hesitant about receiving a COVID-19 vaccine; thus, this hesitancy and lack of trust may be worse for the general public. Third, as previously stated, the survey was conducted in April 2021, which was only a few months into the COVID-19 vaccination process. As the pandemic continues, attitudes may change over time, and future work should seek to re-examine these attitudes at a later point in time. Finally, we were unable to examine political affiliation, which could play a role in individuals’ perceptions about both the vaccine [49] and their trust in institutions [50].

### 4.6. Final Thoughts

It is worrisome that trust in institutions such as government and healthcare is low. In light of these findings, we consider it fortunate that a high percentage of the population has decided to get vaccinated. One major concern stemming from our findings, however, is the question of what happens next time? If an erosion of trust in these institutions has occurred due to inconsistent messaging from officials and widespread mis- and disinformation from alternative sources, will this erosion of trust in our institutions continue? Will we see low compliance with the receipt of recommended COVID-19 booster shots? Will we see downward trends in flu vaccinations in the coming seasons or in children’s immunizations? Perhaps most chilling is the question of what effects this will have on future pandemics and other national emergencies.

## 5. Conclusions

Even among first responders, trust regarding COVID-19 vaccine information is generally low. While those who are unvaccinated tended to have lower trust than those vaccinated, the relatively low trust, even among vaccinated EMS professionals, is concerning. Regardless of vaccination status, individuals most commonly trusted healthcare workers; however, one in five vaccinated, and over half of those unvaccinated, did not trust this source. More concerning about this finding is that the respondents were in the healthcare field themselves. Additionally, trust in the government, media, and one’s employer or community was also low for both vaccinated and unvaccinated groups. To help combat vaccine hesitancy due to low trust, future messaging should be clearer about potential uncertainties as well as indicate what information may be subject to change as the science evolves in real-time. Consistent messaging about the evidence for vaccine safety and effectiveness from a wide variety of sources—including government, politicians across the political spectrum, healthcare providers, and the media—are important as the pandemic continues and would likely increase trust in vaccines.

## Figures and Tables

**Table 1:** Demographic of participants. Abbreviations: HS/GED, high school/General Educational Development; IQR, interquartile range; MIHCP, Mobile Integrated Healthcare or Community Paramedicine.

Characteristic	Statistics (*n* = 2257)
Gender—*n* (%)	
Male	1480 (65.6%)
Female	747 (33.1%)
Missing	30 (1.3%)
Age—*n* (%)	
<28 years	524 (23.2%)
29–38 years	564 (25.0%)
39–50 years	583 (25.8%)
>51 years	585 (25.9%)
Race and Ethnicity—*n* (%)	
White, Non-Hispanic	1910 (84.6%)
Other	269 (11.9%)
Missing	78 (3.5%)
Certification—*n* (%)	
Basic Life Support	900 (39.9%)
Advanced Life Support	1357 (60.1%)
Educational Level—*n* (%)	
HS/GED	211 (9.3%)
Some College	601 (26.6%)
Associate’s	413 (18.3%)
Bachelor’s	508 (22.5%)
Master’s/Doctorate	175 (7.8%)
Urbanicity—*n* (%)	
Urban/suburban	1361 (60.3%)
Rural	793 (35.1%)
Missing	103 (4.6%)
Agency Type—*n* (%)	
Fire	562 (24.9%)
Private	476 (21.1%)
Government, non-fire	293 (13.0%)
Hospital	243 (10.8%)
Other ^1^	201 (8.9%)
Missing	482 (21.4%)
Service Type—*n* (%)	
911	657 (29.1%)
All Others ^2^	272 (12.1%)
Missing	1328 (58.8%)
Years in EMS—mean (IQR)	14.3 (18.0)
Employment Status—*n* (%)	
Full-Time	1265 (56.0%)
Part-Time	212 (9.4%)
Volunteer	240 (10.6%)
Missing	540 (23.9%)

^1^ Other includes air medical, tribal, military, and other; ^2^ Other includes medical transport, 911 and medical transport, clinical services, MIHCP, and other.

## Data Availability

The data presented in this study are available on request from the corresponding author. The data are not publicly available due to participant privacy concerns.
